# Umgang mit subjektiv erlebten Coronarisiken: Sichtweisen junger chronisch kranker Erwachsener

**DOI:** 10.1007/s11553-023-01020-z

**Published:** 2023-03-17

**Authors:** Gundula Röhnsch, Uwe Flick

**Affiliations:** Fachbereich: Erziehungswissenschaften und Psychologie, Arbeitsbereich: Qualitative Sozial- und Bildungsforschung, Freie Universität Berlin, Habelschwerdter Allee 45, 14195 Berlin, Deutschland

**Keywords:** Chronische Erkrankungen, Junge Erwachsene, Qualitative Studie, Coronapandemie, Schutzverhalten, Chronic diseases, Young adults, Qualitative study, COVID-19 pandemic, Protective behavior

## Abstract

**Hintergrund:**

Junge Erwachsene mit chronischen Erkrankungen gelten als Gruppe, die durch Corona gefährdet ist. Ob und wie die Betroffenen sich zu schützen versuchen, welche Risikowahrnehmungen sie verdeutlichen und wie der Lockdown erlebt wird, dazu mangelt es an Erkenntnissen primär im deutschen Sprachraum.

**Ziele und Fragestellung:**

Im Beitrag wird analysiert, welches Coronaschutzverhalten junge chronisch kranke Erwachsene berichten, welche Risikowahrnehmungen sie aufweisen und wie sie den Lockdown erleben.

**Material und Methoden:**

Mit *n* = 59 jungen Erwachsenen (häufig Studierende oder Auszubildende), die von Typ‑1-Diabetes (*n* = 16), Krebs (*n* = 18), chronisch-entzündlichen Darmerkrankungen (*n* = 21) oder von bestimmten seltenen, komplexen Erkrankungen (*n* = 4) betroffen sind, wurden episodische Interviews geführt. Die Datenauswertung erfolgt mittels thematischen Kodierens.

**Ergebnisse:**

Wenige Befragte meinen, durch Corona kaum persönlich gefährdet zu sein, so dass auch Schutzmaßnahmen wie Impfungen sekundär sind. Die meisten Interviewten jedoch berücksichtigen Schutzmaßnahmen penibel. Sie können sich hierzu entweder verpflichtet fühlen, um z. B. andere vulnerable Personen zu schützen, oder sie erleben sich als sehr anfällig für schwere Coronainfektionen. Ungeachtet von erlebten Einschränkungen im Lockdown ist dieser für viele Interviewte auch mit neuen Möglichkeiten verbunden. Online-Formate erleichtern ihnen, Studium/Ausbildung trotz chronischer Erkrankung fortzusetzen und Kontakte mit Freund*innen zu halten.

**Schlussfolgerung:**

Coronarisikowahrnehmungen und Schutzverhalten junger chronisch kranker Erwachsener sollten in ihrem subjektiven Sinn verstärkt in der Versorgung und Begleitung dieser Zielgruppen berücksichtigt werden. Hybride Lehre sollte über den Lockdown hinaus beibehalten werden, damit die jungen Erwachsenen trotz ihrer chronischen Erkrankung sozial teilhaben können und zugleich vor Corona- und weiteren Infektionen geschützt sind.

## Abstract (en)

**Background:**

Young adults with chronic diseases are considered to be at increased risk due to coronavirus disease 2019 (COVID‑19). Information is lacking regarding whether and how they try to protect themselves, what their perception of risk is, and how the lockdown is experienced, in particular in German-speaking areas.

**Objectives:**

The paper analyzes coronavirus protection behaviors of young chronically ill adults, their risk perceptions, and how lockdown was experienced.

**Material and Methods:**

Episodic interviews were conducted with *N* = 59 young adults (mostly students or vocational trainees) affected by type 1 diabetes (*N* = 16), cancer (*N* = 18), inflammatory bowel disease (*N* = 21), or certain rare, complex diseases (*N* = 4). Data analysis is based on thematic coding.

**Results:**

Few respondents believe their personal risk from coronavirus is low, so they consider protective measures such as vaccinations as secondary. However, most interviewees stringently adhere to coronavirus pandemic guidelines. They may either feel obliged to do so, e.g., to protect other vulnerable persons, or they perceive themselves as very susceptible to severe corona infections. Regardless of the experienced limitations during the lockdown, it is also associated with new options for many interviewees. Online teaching makes it easier for them to continue their studies/vocational training despite their chronic illness and to maintain contact with their friends.

**Conclusion:**

Subjective corona risk perceptions and protective behaviors of young chronically ill adults should be considered more in the care and support of these target groups. Hybrid teaching should be continued beyond the lockdown so that young adults can participate socially despite their chronic illness while being protected from coronavirus and other infections.

## Hintergrund

Das Spektrum von Symptomen infolge einer Coronavirusinfektion ist breit gefächert und reicht von Erkältungsanzeichen bis hin zu schweren Lungenentzündungen und Multiorganversagen. Von einem schweren Krankheitsverlauf sind insbesondere (ältere) Menschen mit Vorerkrankungen betroffen [21]. Chronische Erkrankungen können in jedem Lebensalter auftreten, ihre Häufigkeit nimmt weltweit auch im Kindes‑, Jugend- und mithin auch im jungen Erwachsenenalter zu [24]. Inwieweit junge chronisch erkrankte Erwachsene Corona als persönliches Risiko erleben und wie sie damit umgehen, dazu liegen im deutschsprachigen Raum kaum Erkenntnisse vor. Wie junge chronisch erkrankte Erwachsene mit Coronarisiken umgehen, lässt sich angesichts möglicher Auswirkungen einer Infektion auf den Verlauf der chronischen Krankheit auch unter tertiärpräventiven Aspekten [15] mit dem Ziel einer Verhinderung von Krankheitskomplikationen adressieren.

Menschen mit chronischen Erkrankungen stehen vor der Herausforderung, ihre Erkrankung in den Alltag zu integrieren, um ein subjektiv sinnvolles Leben führen zu können [1]. Den gewohnten Alltag aufrechtzuerhalten, hat für Betroffene – insbesondere in Phasen bedingten Gesundseins (vgl. [36]) – oft einen höheren Stellenwert, als sich auf die Erkrankung zu konzentrieren [2]. Gleichwohl müssen chronisch erkrankte Menschen Strategien entwickeln, um z. B. krankheitsbezogene und alltägliche Aufgaben und Anforderungen zu bewältigen, mit Unsicherheiten und Mehrdeutigkeiten zu leben oder um sich durch das Versorgungssystem zu bewegen. Mitarbeit am Versorgungsgeschehen und somit die Übernahme von Verantwortung für die eigene (verbliebene) Gesundheit wird von chronisch kranken Menschen geradezu erwartet (vgl. [14, 19, 37]). In Gesundheitspolitik und -versorgung gelten diese insofern nicht länger als „passiv erduldende“ Patient*innen, sondern als Koproduzent*innen von Gesundheit [3]. Ein hiermit zusammenhängendes (Ideal)bild von mündigen Bürger*innen/Patient*innen, die sich im Umgang mit ihrer Erkrankung „expertisiert“ haben (vgl. [23, 30]), steht jedoch auch unter Kritik: Dies, weil es auf der Vorstellung eines rationalen und logischen Handelns fußt, in dessen Rahmen bewusste Entscheidungen für oder gegen gesundheitsbezogene Verhaltensalternativen fallen [30]. Subjektive Bedeutung und Erfahrungen chronischer Krankheiten werden ebenso ausgeklammert wie kulturelle Normen und Gewohnheiten oder lebensweltliche Kontexte, die die Bewältigung von chronischer Krankheit fördern oder begrenzen [14, 30]. Ausgeblendet wird insbesondere, dass Menschen aus prekären sozialen Verhältnissen (vgl. [19]) sowie Menschen, die in bestimmten Phasen ihrer chronischen Erkrankung bzw. ihres Versorgungsverlaufs hoch belastet sind (vgl. [34]), wenig Ressourcen haben, sich aktiv um die eigene Gesundheit zu kümmern.

Auch in der Coronapandemie zielen Regierungen und Gesundheitsbehörden auf individuelle Verantwortung, wenn sie appellieren, keine unnötigen Risiken einzugehen, die zur weiteren Verbreitung des Virus beitragen [31]. Allerdings belegen Studien ein Sinken der Motivation, Schutzmaßnahmen zu berücksichtigen, speziell unter jungen Erwachsenen [6]. Während diese Coronainfektionen oft aufgrund ihres Alters für unbedenklich halten [40], gelten sie als besonders vulnerabel für emotionalen Stress infolge des Corona-Lockdowns [17]. Speziell Studierende fühlen sich unsicher, was Zukunfts- und Bildungsperspektiven anbelangt. Zudem erleben sie den plötzlichen Übergang vom Präsenzunterricht zur Online-Lehre als herausfordernd. Für sie ist damit eine neue Form des Lernens verbunden, die technische Fähigkeiten und Ausstattungen ebenso erfordert wie die Anpassung an ein neues, oft inadäquates Lernumfeld [17].

Insbesondere im deutschsprachigen Raum nehmen Studien zur Situation von jungen – studierenden – Erwachsenen in der Coronapandemie selten auf sozial oder gesundheitlich vulnerable Subgruppen Bezug. Zu diesen kaum berücksichtigten Gruppen gehören junge Erwachsene mit u. a. körperlichen Beeinträchtigungen [28] wie chronischen Erkrankungen. Internationale Studien zeichnen kein einheitliches Bild zu Risikowahrnehmungen junger chronisch kranker Erwachsener. So finden sich für junge Erwachsene mit Diabetes Belege, dass diese Coronarisiken ebenso wie die Bedeutung präventiver Maßnahmen eher unterschätzen [32], während junge Krebspatient*innen schwerwiegende Konsequenzen einer Coronainfektion befürchten [7].

Für junge chronisch kranke Erwachsene ist die Pandemie möglicherweise besonders herausfordernd, weil es ihnen oft ohnehin schwerfällt, tragfähige Gleichaltrigenbeziehungen aufzubauen und zu halten [20, 33], was ihr Risiko zu vereinsamen erhöht [25].

Ausgehend von einer eigenen Studie greift der vorliegende Beitrag die im deutschsprachigen Raum bestehende Forschungslücke zu jungen chronisch kranken Erwachsenen auf: Er geht den Fragen nach, a) welches Coronaschutzverhalten diese auf der Basis welcher Risikowahrnehmungen aufweisen und wie sich Schutzverhalten im Kontext des alltäglichen Umgangs mit chronischer Erkrankung darstellt und b) wie der Lockdown erlebt wird und welche Herausforderungen sich hier potenziell ergeben.

## Methoden

Die dem Artikel zugrunde liegende qualitative Studie untersucht, wie chronisch erkrankte junge Erwachsene Gleichaltrigenbeziehungen aufbauen und pflegen und welche Bedeutung diese für sie haben. Dazu werden primär junge Erwachsene mit einer der folgenden Erkrankungen berücksichtigt: a) chronisch-entzündliche Darmerkrankungen (CED), b) Diabetes mellitus/I, c) Krebserkrankung. Ergänzend werden einige junge Erwachsene mit bestimmten seltenen, komplexen Erkrankungen in die Studie einbezogen, die eine ungünstige bzw. unklare Prognose haben (Mukoviszidose, terminale Niereninsuffizienz). Studienteilnehmer*innen sind nicht nur die jungen Erwachsenen selbst, sondern auch deren engen Freund*innen/Partner*innen^1^.

### Feldzugang

Aus Gründen des Infektionsschutzes erfolgte der Feldzugang ausschließlich digital. Zunächst wurden Selbsthilfegruppen, Patientenverbände, Beratungsstellen, Kliniken und Projekte recherchiert, die junge Erwachsene mit chronischen Erkrankungen erreichen. Entscheidungsträger*innen dieser Organisationen und Einrichtungen wurden über die Studienhintergründe informiert und gebeten, Flyer und Studieninformationen an junge Erwachsene weiterzuleiten. Bei Interesse vereinbarten diese einen Interviewtermin mit der Erstautorin. Vor Gesprächsbeginn wurden sie nochmals persönlich über wesentliche Interviewthemen informiert und konnten offene Fragen zur Studie klären.

### Studiensample

Als Studienteilnehmende wurden *n* = 59 chronisch kranke junge Erwachsene – 43 Frauen und 16 Männer – im Alter zwischen 18 und 32 Jahren rekrutiert. Sie verteilen sich wie folgt (Tab. 1) auf die Krankheitsgruppen.

**Tab. 1:** Studiensample – Alter und Krankheitsgruppen

Alter	Krankheitsgruppe					
	Diabetes	Krebs	Chronisch-entzündliche Darmerkrankungen (CED)	Seltene, komplexe Erkrankungen		Gesamt
				Mukoviszidose	Terminale Niereninsuffizienz	
	*n* = 16	*n* = 18	*n* = 21	*n* =2	*n* =2	*n* = 59
Bis 20 Jahre	2	4	4	–	–	10
21–25 Jahre	11	6	9	1	–	27
26–30 Jahre	3	7	8	1	2	21
Älter als 30 Jahre	–	1	–	–	–	1

Das Durchschnittsalter der Befragten liegt bei 25,7 Jahren. Etwa zwei Drittel der Interviewten (*n* = 39) sind Studierende oder Auszubildende.

### Datenerhebung und -auswertung

Mit den jungen Erwachsenen wurden episodische (Leitfaden)interviews [13] geführt, die die Prinzipien von Erzählung und Befragung kombinieren. Zunächst sollten die Interviewten ihre themenbezogenen Erfahrungen eher allgemein darstellen. Im Weiteren wurden sie gebeten, diesen Erfahrungen zugrundeliegende Situationen (Episoden) zu erzählen.

Die Interviews wurden von November 2021 bis August 2022 von der Erstautorin durchgeführt und dauerten durchschnittlich 75 min. Schwerpunkte des Leitfadens waren: Krankheitserleben, „Disclosure“, Selbstmanagement, soziale Unterstützung, soziale Beziehungen im Krankheitsverlauf, Freundschaftsrepräsentationen.

Mittels Webex-Videokonferenz fanden 52 Interviews statt, lediglich 7 Befragte bevorzugten aus persönlichen Gründen ein Telefoninterview. Nach Beendigung des Interviews wurden alle Studienteilnehmenden gefragt, wie es ihnen im Interviewverlauf „ergangen“ ist, um das Gespräch emotional stützend abzuschließen.

Die Auswertung der Interviews erfolgte unter Anwendung von MAXQDA (Version 22.0.1, VERBI GmbH, Berlin) mittels thematischen Kodierens [13]. Zunächst wurden alle Aussagen zu einem Themenbereich fallbezogen kodiert [38] und kategorisiert. Um eine fallübergreifende thematische Struktur zu entwickeln, wurden die Kategorien anschließend zwischen den einbezogenen Fällen abgeglichen. An die Herausarbeitung dieser thematischen Struktur schloss sich eine Feinanalyse der einzelnen Kategorien an. Dazu wurden unter Anwendung des Strauss’schen Kodierparadigmas [38] ausgewählte, den Kategorien zugeordnete Textpassagen in ihrem Sinngehalt interpretiert. Um alternative Lesarten zu berücksichtigen, wurden diese Interpretationen ausführlich im Team diskutiert.

## Ergebnisse

Im Folgenden wird analysiert, welches Coronaschutzverhalten die chronisch kranken jungen Erwachsenen berichten und welche Risikowahrnehmungen und subjektiven Überzeugungen sie in dem Kontext verdeutlichen. Anschließend ist von Interesse, wie junge chronisch erkrankte Erwachsene den Lockdown wahrnehmen. Die Aussagen der Befragten lassen sich verschiedenen thematischen Gruppen zuordnen.

### Umgang mit Corona-bezogenen Risiken

#### Sich kaum betroffen fühlen

Die Interviewten aus einer ersten Gruppe verbindet, dass eine Infektion mit dem Coronavirus als nebensächlich gesehen wird. Während diese Befragten allgemeine Coronaschutzmaßnahmen je nach Situation einhalten, verzichten sie auf die Inanspruchnahme von Impfungen gegen COVID-19 („coronavirus disease 2019“).

Abgesehen von einer Befragten, fühlen sich diese Interviewten subjektiv gesund. Sie halten eine gesunde Lebensweise für wichtig, auch, weil sie so ihre chronische Erkrankung unter Kontrolle halten können. Da es diesen Interviewten subjektiv gesundheitlich gut geht, sehen sie sich weder als besonders anfällig für eine Coronainfektion an, noch fürchten sie, dass diese schwer verlaufen oder sich ungünstig auf ihre chronische Grunderkrankung auswirken könnte. Corona erscheint so als Thema, das sie nicht betrifft und mit dem sie sich nicht auseinandersetzen müssen. Diese Interviewten ignorieren jedoch, dass bereits Komplikationen ihrer chronischen Erkrankung eingetreten sind. Unter Verweis darauf, i. Allg. gesundheitsbewusst zu sein, blenden sie aus, dass sie unter Pandemiebedingungen und damit verbundenen Homeoffice-Regelungen, an die sie sich erst gewöhnen müssen, ein regelmäßiges Management ihrer chronischen Erkrankung vernachlässigen.

„Ich achte auf mich, und dadurch, dass ich einen sehr gesunden Lebensstil habe und auch sportlich mich sogar täglich betätige (…) ich war [während des Lockdowns – die Verf.] mehr oder weniger zu 70 % mehr daheim (…) dann kontrolliere ich weniger und messe weniger.“ (Justus^2^, 343–345)

Anders als Interviewte, die sich für gesund halten und sich daher nicht näher mit Corona befassen, verdeutlicht eine Befragte aus dieser Gruppe, dass sich eine mögliche Coronainfektion für sie stark relativiert, *weil* sie eine chronische Erkrankung hat. In deren Verlauf wurde sie mehrfach mit der eigenen Endlichkeit konfrontiert. Ein mögliches baldiges Lebensende – egal, ob infolge einer Coronainfektion oder aufgrund der chronischen Erkrankung – ist für diese Befragte etwas, das sie kaum noch überraschen kann, das daher auch nur wenig angstauslösend bzw. handlungsrelevant ist, um eine Infektion verhindern zu wollen.

„(…) ich hab’ auch keine Angst zu sterben (…) ich bin so oft jetzt schon damit konfrontiert gewesen (…) ich werde den Zeitpunkt sowieso nicht bestimmen“. (Antonia, 398)

#### Sich verpflichtet sehen

Befragte aus einer zweiten Gruppe fühlen sich zu Coronaschutzmaßnahmen nahezu verpflichtet. Sie gehen allgemein davon aus, dass Coronainfektionen ernstzunehmende Erkrankungen darstellen. Dies allein rechtfertigt es oft bereits, sich impfen zu lassen. Hierauf zu verzichten, wäre aus Sicht dieser Interviewten tendenziell fahrlässig – unabhängig davon, welches Risiko sie persönlich mit der Nicht-Inanspruchnahme von Impfungen eingehen würden. In der Annahme, dass Coronainfektionen eine – nicht näher spezifizierte – Gesundheitsgefahr darstellen, sind Impfungen für diese Befragten eine normative Selbstverständlichkeit.

„Und für mich steht nie in Frage, mich nicht impfen zu lassen. Das ist für mich gar nicht so, dass ich jetzt über eine Impfung nachdenke (…). Wenn ich mich nicht impfen lasse, sterbe ich (…) das könnte gefährlich sein (…). Ich sehe es eher so, Impfen gehört sich. Wer sich nicht impft, geht ein Risiko ein.“ (Jonah, 262)

Einige Interviewte aus dieser Gruppe verdeutlichen, gegenwärtig in einem stabilen Stadium der chronischen Erkrankung zu sein, so dass sie z. B. keine immunsuppressive Behandlung bekommen. Ihnen ist daher unklar, ob sie selbst ein hohes Risiko haben, sich mit dem Coronavirus zu infizieren und einen schweren Krankheitsverlauf zu erleiden. Dass dies sehr wohl der Fall sein könnte, dafür werden sie erst durch Freund*innen oder Ärzte/Ärztinnen sensibilisiert. Womöglich selbst zu einer Coronarisikogruppe zu gehören, stellt für die Befragten eine neue, potenziell verunsichernde Erfahrung dar. Zwar ist für sie eine solche Fremdeinschätzung „objektiv“ gerechtfertigt, sie steht jedoch im Widerspruch zu ihrer gesundheitsbezogenen Selbstwahrnehmung.

Da es diese Interviewten letztlich nicht ausschließen, für – schwere – Coronainfektionen anfällig zu sein, aber auch, weil es Bezugspersonen von ihnen implizit erwarten, nehmen sie Schutzimpfungen gegen COVID-19 zeitnah in Anspruch. Hierzu fühlen sich diese Befragten auch verpflichtet, weil sie chronisch krank sind.

„(…) weil ich (…) auch von außen darauf hingewiesen wurde ‚Du, wie sieht’s aus mit der Impfung? Du vielleicht auch ein bisschen früher als andere?‘ (…) da hab’ ich auch noch mal so ’n bisschen, ja, nicht unbedingt Angst entwickelt, aber schon so auch einen Blick dafür (…) generell chronische Krankheiten (…) diese Gruppen müssen irgendwie noch mal so ’n bisschen mehr auf sich achten.“ (Ina, 211)

In der Verantwortung sehen sich diese Befragten nicht nur für die eigene Person, sondern auch für ihre Angehörigen sowie für Personen aus ihrem sozialen Umfeld, die sie für mindestens ebenso anfällig halten wie sich selbst. Für diese Interviewten ist der Gedanke belastend, diese anderen im Fall einer Coronainfektion womöglich anzustecken. Vor allem, um dies zu verhindern, aber auch aus Gründen des Selbstschutzes belassen sie es nicht dabei, sich gegen Corona impfen zu lassen, sondern berücksichtigen die geltenden Coronaregelungen sehr genau. Sensibilisiert durch ihre (schwere) Erkrankung, legen sie diese häufig deutlich strenger aus als „vorgeschrieben“. Wenn sie dazu z. B. auf das Zusammensein mit Freund*innen verzichten, die ihrer Meinung nach weniger sorgfältig sind in der Einhaltung von Schutzmaßnahmen, riskieren sie Auseinandersetzungen. Dies nehmen sie hin, ohne Kompromisse beim Infektionsschutz einzugehen – zu stark ist die subjektiv erlebte Verantwortlichkeit gegenüber infektionsgefährdeten Personen ihres Umfelds.

„(…) dass ich in der Coronapandemie vorsichtiger (…) bin, weil ich weiß, wie es ist, wenn man schwer erkrankt oder jemanden auch sterben sieht (…) habe ich schon das Gefühl, das häufiger mal erklären zu müssen, aber auch dann, also spätestens nach der Erklärung ist eigentlich das dann klar.“ (Friederike, 202)

#### Sich als hochgradig gefährdet ansehen

Wie den Befragten aus der soeben dargestellten Gruppe ist auch Interviewten in einer dritten Gruppe die Einhaltung von Coronaschutzmaßnahmen sehr wichtig – allerdings nicht aufgrund normativen oder sozial-moralischen Verpflichtet-Seins. Vielmehr erleben sich die hier kategorisierten Befragten als stark gefährdet sowohl für eine Infektion mit dem Coronavirus als auch für einen schweren Infektionsverlauf. Zudem gehen sie davon aus, dass es im Fall einer Infektion zu schweren Komplikationen ihrer chronischen Grunderkrankung kommt. Dies löst bei ihnen große Ängste aus.

„(…) das war wie so ’ne Art Kurzschluss (…) wenn ich das [Corona – die Verf.] kriege, werde ich daran sterben, weil ich eine Erkrankung der Lunge habe.“ (Matteo, 10)

Wie diese Interviewten verdeutlichen, haben sie sich intensiv mit dem Thema „Corona“ auseinandergesetzt. Sie haben sich im Internet informiert, Studien gelesen und mit Ärzten/Ärztinnen gesprochen, um ihr persönliches Coronarisiko einschätzen zu können. Für sie ist es von höchster Priorität, eine Infektion zu vermeiden. Wenn auch mit unterschiedlicher Intensität ordnen sie dem ihre alltäglichen Verpflichtungen sowie Kontakte zu Freund*innen unter. So verzichten sie auf gemeinsame Freizeitaktivitäten – erst recht, wenn Freund*innen Coronaregelungen offenbar nicht ausreichend ernstnehmen – und riskieren, von diesen künftig abgelehnt zu werden. Einige dieser Befragten versuchen sich komplett aus dem sozialen Leben zurückzuziehen, was zu starken Beeinträchtigungen ihres psychosozialen Wohlbefindens führt. Zudem erwarten sie von Freund*innen und weiteren Personen des sozialen Umfelds, dass sie Schutzmaßnahmen ihnen zuliebe genau einhalten. Sind diese dazu nur partiell bereit, wird das als Rücksichtslosigkeit erlebt. Wenn die Anderen zur potenziellen „Gefahr“ für die eigene Gesundheit werden, löst das Ängste und Verbitterung aus.

„(…) ich unterstelle einfach (…) jeder Person, die keine Maske trägt, dass sie mich umbringen will, also dass ihr das wirklich egal ist, ob sie andere schützt oder nicht.“ (Jannis, 386)

Die verschiedenen Gruppen von Aussagen, denen sich die Befragten zuordnen lassen, werden abschließend tabellarisch zusammengefasst, bevor darauf eingegangen wird, wie der Lockdown wahrgenommen wird (Tab. 2).

**Tab. 2:** Risikowahrnehmungen bei Interviewten der einzelnen Krankheitsgruppen

Muster	Gesamt	Diabetes	Krebs	Chronisch-entzündliche Darmerkrankungen (CED)	Seltene, komplexe Erkrankungen	
					Mukoviszidose	Terminale Niereninsuffizienz
Sich kaum betroffen fühlen	8	3	2	2	–	1
Sich verpflichtet sehen	11	5	2	3	–	1
Sich als hochgradig gefährdet ansehen	40	8	14	16	2	–
*n* =	59	16	18	21	2	2

Wie sich – analog zu den Ergebnissen internationaler Studien [7, 32] – zeigt, sind es tendenziell eher junge Erwachsene mit Diabetes, die sich als wenig betroffen durch Corona ansehen, sowie junge Erwachsene mit schwereren Erkrankungen wie Krebs oder chronisch-entzündlichen Darmerkrankungen (CED), die sich als sehr anfällig erleben (Tab. 2).

### Wahrnehmung des Lockdowns

Nur wenige Interviewte zweifeln an Coronaregelungen. Die weitaus meisten Befragten akzeptieren präventive Maßnahmen wie die Reduktion von Sozialkontakten oder das Tragen von Masken als etwas, mit dem sie im Verlauf der Behandlung ihrer chronischen Erkrankung ohnehin konfrontiert wurden. Akzeptiert wird auch der Lockdown als Schutz vor einer Coronainfektion. Gleichwohl ist er für die Interviewten mit neuen Möglichkeiten ebenso wie mit Herausforderungen verbunden.

#### Neue Möglichkeiten

Interviewte, die dem Lockdown Positives abgewinnen, verweisen auf (Lehr)veranstaltungen und private Begegnungen in dieser Zeit, die online stattfinden. Online-Kontakte erlauben den Befragten, auch in Phasen eines krisenhaft zugespitzten Verlaufs ihrer chronischen Erkrankung sozial teilzuhaben. So gelingt es ihnen dank sozialer Medien, mit Freund*innen verbunden zu bleiben und gemeinsam neue Formen der Freizeitgestaltung zu entdecken, wenn sie direkte soziale Kontakte meiden müssen. Online-Formate ermöglichen es jedoch v. a., berufliche Abschlüsse zu erreichen und das Studium fortzusetzen. Präsenzveranstaltungen bringen die Interviewten an Grenzen, wenn es ihnen krankheitsbedingt schlecht geht.

„(…) nicht geschafft, 20 km weiter zur Hochschule zu fahren oder da auch zu sitzen. Ich habe mir die Vorlesungen online im Bett oft angehört und konnte es mitverfolgen (…) wenn es nicht online gewesen wäre, hätte ich das Studium abbrechen müssen.“ (Leah, 227)

Die Befragten meinen zudem, dass sie im Homeoffice mehr Zeit für das „Management“ ihrer chronischen Erkrankung haben und flexibler auf gesundheitliche Bedürfnisse reagieren können.

Aus Sicht einiger Befragter führt der Lockdown auch dazu, dass sich der Alltag von „gesunden“ Gleichaltrigen ihrem eigenen angleicht. Es entlastet sie zu sehen, dass nicht nur sie selbst angesichts ihrer chronischen Erkrankung, sondern auch Freund*innen aufgrund des Lockdowns an sozialen Aktivitäten wie Gaststätten- oder Konzertbesuchen gehindert sind. Das Erleben, dass auch die Gleichaltrigen zurückstecken müssen, erleichtert es den Interviewten, ihre Erkrankung zu akzeptieren. Sie fühlen sich weniger sozial ausgeschlossen.

„(…) das [der Lockdown – die Verf.] war ja so eine kollektive Erfahrung, und als ich krank war, war ich die Einzige, und alle haben einfach weitergemacht mit ihrem Studium, ihren Ausbildungen (…) haben ihre Leben gelebt, und ich war die einzige, die sozusagen abgeschnitten war.“ (Pauline, 364)

#### Herausforderungen

Befragte, die den Lockdown (auch) als herausfordernd wahrnehmen, verweisen auf die mit den Schutzmaßnahmen verbundenen Restriktionen. Diese verschärfen in ihren Augen solche Einschränkungen, mit denen sie bislang krankheitsbedingt konfrontiert waren, wenn sie etwa aufgrund anstrengender Therapien das Haus nicht mehr verlassen konnten. Zudem beklagen die Interviewten begrenzte Möglichkeiten, gemeinsam mit Freund*innen die Freizeit zu verbringen. Während Freundschaften an Nähe verlieren, mangelt es an Gelegenheiten, neue soziale Kontakte aufzubauen. Wenn sich das soziale Netz der Befragten ausdünnt, bedeutet dies für sie einen Verlust an Lebensqualität und an Ressourcen im Umgang mit ihrer chronischen Erkrankung, denn „(…) menschliche Nähe ist das Einzige, was einen so’n bisschen von so einer Krankheit auffangen kann“ (Joshua, 363).

## Diskussion

Ziel dieses Beitrags ist es zu analysieren, a) welches Coronaschutzverhalten die jungen Erwachsenen berichten, wie sich dieses in den Umgang mit ihrer chronischen Grunderkrankung einreiht und welche Risikowahrnehmungen und sonstigen subjektiven Überzeugungen sie in dem Kontext deutlich machen und b) wie der Lockdown wahrgenommen wird.

Wie die Ergebnisse unserer Studie verdeutlichen, fühlen sich einige wenige Interviewte vom Risiko einer Coronainfektion wenig betroffen. Unter Verweis auf ihre insgesamt gesunde Lebensweise sehen sie von Coronaschutzmaßnahmen wie Impfungen ab. Wenn sie versuchen, sich durch ihren pandemiebedingt veränderten Alltag „durchzuwursteln“ [4], füllt sie dies so aus, dass sie ein regelmäßiges Management ihrer chronischen Erkrankung oft vernachlässigen (vgl. [2]).

Unsere Studienergebnisse zeigen jedoch auch, dass die Befragten mehrheitlich Schutzmaßnahmen für sehr wichtig halten, sie im Alltag penibel berücksichtigen und häufig strenger auslegen als gesellschaftlich gefordert. Im Fall dieser Interviewten wird deutlich, wie stark der sozial- und versorgungswissenschaftliche Diskurs um eine Verantwortungsübergabe von Gesundheit an chronisch erkrankte Menschen selbst (vgl. [14, 19, 37]) in das soziale Leben der Betroffenen eingedrungen ist. So sieht sich ein Teil dieser Befragten zu Coronaschutzverhalten regelrecht verpflichtet – unter abstrakt-normativen Aspekten, aber auch, um vulnerable Angehörige bzw. Bekannte oder sich selbst als chronisch Erkrankte*n vor Infektionen zu bewahren. Vergleichbar zu Ergebnissen anderer Studien [18, 39] erleben sich diese Interviewten vorrangig aufgrund äußerer Zuschreibungen als anfällig für schwere Infektionen. Individuelles Schutzverhalten orientiert sich in dem Kontext auch an den (vermeintlichen) Erwartungen dieser anderen Personen.

Ein hohes Maß an Verantwortungsübernahme für die eigene Gesundheit demonstrieren auch Interviewte, die sich intensiv mit Coronarisiken auseinandergesetzt haben und sich als hochgradig gefährdet erleben. Infektionen wollen sie daher unbedingt vermeiden. Wenn sich diese Befragten im Umgang mit Corona partiell „expertisiert“ zu haben scheinen (vgl. [23, 30]), zahlen sie einen hohen Preis: Zum einen setzen sie bestehende Freundschaften aufs Spiel, wenn sie auf der Einhaltung von Schutzmaßnahmen bestehen – dies vor dem Hintergrund, dass es jungen chronisch kranken Erwachsenen krankheitsbedingt oft schwerfällt, neue Freundschaften zu knüpfen [20, 33]. Zum anderen wird in ihrem Fall deutlich, analog zu Studienergebnissen zum Umgang mit Infektionsrisiken in anderen Kontexten (vgl. [27]), wie der auf Corona bezogene Risikodiskurs zu moralischen Aufladungen beitragen und moralischer Panik führen kann. So ist unseren Befragten bewusst, dass freiwillige Selbstisolation aus Gründen des Infektionsschutzes zu Lasten ihres Wohlbefindens geht. Zugleich jedoch identifizieren sie Gleichaltrige, die Coronaregeln weniger streng beachten, als rücksichtslos oder gar gefährlich.

Im Ergebnis unserer Studie wird auch deutlich, dass der Lockdown für die meisten Befragten keine besondere Belastung darstellt – deshalb nicht, weil sie es gewohnt sind, sich phasenweise selbst zu isolieren oder Masken zu tragen (z. B. im Rahmen einer immunsuppressiven Behandlung/Chemotherapie). Die Interviewten verfügen somit über Ressourcen im Umgang mit Lockdown-bedingten Herausforderungen, wie sie ansonsten für alte (chronisch kranke) Menschen beschrieben werden, die im Laufe ihres Lebens wiederholt gesundheitliche oder gesellschaftliche Krisen bewältigen mussten [26, 42].

Die meisten unserer Befragten verweisen sogar auf Lockdown-bedingte Entlastungen. So sehen sie Online-Lehre – anders als Studierende der ‚Durchschnittsbevölkerung‘, die zu dieser zumindest zu Pandemiebeginn oft kritisch eingestellt waren [9, 17] – häufig als Voraussetzung, um trotz chronischer Krankheit ihr Studium fortsetzen und mit Freund*innen in Kontakt bleiben zu können.

## Ausblick

Junge chronisch kranke Erwachsene sollten als Expert*innen für den alltäglichen Umgang mit ihrer Erkrankung verstanden werden, die auf Grundlage krankheitsspezifischen Alltagswissens und geprägt durch ihre soziokulturelle Lebenswelt (potenziell) kompetent handeln (vgl. [10, 29, 41]) – auch im Umgang mit Coronarisiken. Um sie hierin zu unterstützen, sollten Corona-bezogene Risikowahrnehmungen und Schutzverhaltensweisen in der Versorgung und psychosozialen Begleitung junger chronisch kranker Erwachsener verstärkt evaluiert und berücksichtigt werden. Jungen Erwachsenen, die meinen, dass Corona kein Thema für sie ist, sollte ein stärkeres Gefühl möglichen eigenen Betroffenseins vermittelt werden. Dazu sollten sie sachbezogene Informationen zu Coronarisiken im Kontext ihrer Krankheit erhalten – ebenso wie chronisch kranke junge Erwachsene, die starke, nahezu panische Ängste angesichts einer möglichen Infektion entwickeln.

Wiederum sollte die Tatsache verstärkt gewürdigt werden, dass junge chronisch kranke Erwachsene den Lockdown oft differenziert wahrnehmen und kaum unter beeinträchtigtem Wohlbefinden leiden. Solch eine psychische Stabilität lässt sich als Ressource verstehen (vgl. [42]). Als solches sollte sie in die Ausgestaltung von Unterstützungsangeboten für „gesunde“ Gleichaltrige, die unter den Folgen des Lockdowns leiden, einbezogen werden. Junge chronisch kranke Erwachsene könnten sich im Gegenzug in ihrer Stärke anerkannt und wertgeschätzt fühlen.

## Limitationen

Ziel dieser Studie ist nicht die repräsentative Erfassung von Coronarisikobewusstsein, sondern die Darstellung von subjektiven Coronaschutzverhaltensweisen sowie von Risikowahrnehmungen junger chronisch kranker Erwachsener. Mithin werden – potenzielle – Risikogruppen fokussiert, deren subjektiven Sichten zu Corona in bisherigen Studien kaum berücksichtigt wurden [28].

Unsere Studie hat einige Limitationen:

1. Das Sample besteht primär aus jungen Erwachsenen, die ein Studium absolvieren oder kürzlich abgeschlossen haben. Selten gelang es, Personen mit niedrigem Bildungsniveau in die Studie einzubeziehen, die oft kritischer gegenüber allgemeinen [35] oder speziellen Coronaschutzmaßnahmen wie Impfungen [12] eingestellt sind.
2. Junge Frauen sind im Sample in der Überzahl – was sich ähnlich auch in anderen qualitativen [11, 16] und quantitativen [8, 33] Studien zu chronisch kranken jungen Erwachsenen zeigt. Bezogen jedoch speziell auf Corona, verdeutlichen vorliegende Studien, dass Frauen eher als Männer Coronaschutzmaßnahmen einhalten [5, 22]. Dies könnte die in unserer Studie identifizierte insgesamt hohe Schutzbereitschaft miterklären.
3. Das Sample umfasst überwiegend junge Erwachsene mit schweren chronischen Erkrankungen bzw. Krankheitsverläufen. Inwieweit sich die in unserer Studie identifizierte besondere Sensibilität gegenüber Coronarisiken und die ausgeprägte Schutzbereitschaft auch bei jungen Erwachsenen mit leichteren chronischen Erkrankungen finden lassen, ist unklar und bedarf weiterer Forschung.

## Fazit für die Praxis

- Junge chronisch kranke Erwachsene setzen sich oft intensiv mit Coronarisiken auseinander und entwickeln auf dieser Basis das Bedürfnis, sich zu schützen. Ihre Risikowahrnehmungen sollten in Prävention und Versorgung sowie in der Coronapolitik vermehrt berücksichtigt werden.
- Die Fähigkeit vieler chronisch kranker junger Erwachsener, sich mit Lockdown-bedingten Herausforderungen zu arrangieren, sollte als Stärke anerkannt und in die Ausgestaltung von Maßnahmen zur Prävention und Bewältigung von Corona-bezogenem Distress, den Gleichaltrige oft empfinden, einbezogen werden.
- Angesichts der großen Bedeutung von Online-(Lehr‑)Angeboten für junge chronisch kranke Erwachsene sollten im Bildungssystem auch bei Präsenzbetrieb verstärkt hybride Lehrangebote vorgehalten werden.

## Footnotes

1 Da dieser Artikel ausschließlich auf chronisch kranke junge Erwachsene als „Coronarisikogruppe“ Bezug nimmt, beziehen sich die methodischen Ausführungen einzig auf dieses Sample und nicht auf das der Peers.

2 Alle Namen pseudonymisiert.
